# Inference following multiple imputation for generalized additive models: an investigation of the median *p*-value rule with applications to the Pulmonary Hypertension Association Registry and Colorado COVID-19 hospitalization data

**DOI:** 10.1186/s12874-022-01613-w

**Published:** 2022-05-21

**Authors:** Matthew A. Bolt, Samantha MaWhinney, Jack W. Pattee, Kristine M. Erlandson, David B. Badesch, Ryan A. Peterson

**Affiliations:** 1 Department of Biostatistics and Informatics, Colorado School of Public Health, University of Colorado-Denver Anschutz Medical Campus, 13001 E. 17th Pl, Aurora, CO USA; 2 School of Medicine, University of Colorado-Denver Anschutz Medical Campus, Aurora, CO USA

**Keywords:** Missing data, Pooling results, Simulation study, Splines, Elevation, Pulmonary arterial hypertension, COVID-19, Cauchy combination test

## Abstract

**Background:**

Missing data prove troublesome in data analysis; at best they reduce a study’s statistical power and at worst they induce bias in parameter estimates. Multiple imputation via chained equations is a popular technique for dealing with missing data. However, techniques for combining and pooling results from fitted generalized additive models (GAMs) after multiple imputation have not been well explored.

**Methods:**

We simulated missing data under MCAR, MAR, and MNAR frameworks and utilized random forest and predictive mean matching imputation to investigate a variety of rules for combining GAMs after multiple imputation with binary and normally distributed outcomes. We compared multiple pooling procedures including the “D2” method, the Cauchy combination test, and the median *p*-value (MPV) rule. The MPV rule involves simply computing and reporting the median *p*-value across all imputations. Other ad hoc methods such as a mean *p*-value rule and a single imputation method are investigated. The viability of these methods in pooling results from B-splines is also examined for normal outcomes. An application of these various pooling techniques is then performed on two case studies, one which examines the effect of elevation on a six-minute walk distance (a normal outcome) for patients with pulmonary arterial hypertension, and the other which examines risk factors for intubation in hospitalized COVID-19 patients (a dichotomous outcome).

**Results:**

In comparison to the results from generalized additive models fit on full datasets, the median *p*-value rule performs as well as if not better than the other methods examined. In situations where the alternative hypothesis is true, the Cauchy combination test appears overpowered and alternative methods appear underpowered, while the median *p*-value rule yields results similar to those from analyses of complete data.

**Conclusions:**

For pooling results after fitting GAMs to multiply imputed datasets, the median *p*-value is a simple yet useful approach which balances both power to detect important associations and control of Type I errors.

**Supplementary Information:**

The online version contains supplementary material available at 10.1186/s12874-022-01613-w.

## Background

Before the onset of data collection in many studies, sample size calculations are conducted to ensure that the study is adequately powered to find a meaningful clinical effect size if one exists. In all but the rarest circumstances, some data cannot be collected as planned, which leaves the analyst with a dataset that is either underpowered, biased in some manner, or both. Typically, missing data are categorized into three common patterns. Datasets with missingness completely at random (MCAR) occur when the probability of a data point being missing is completely independent of its value or the value of other variables. Missingness at random (MAR) instead refers to when the probability of a value being missing is dependent on other covariates in the dataset, but not dependent on the true value of the variable. The most difficult missing data pattern, missingness not at random (MNAR), occurs when the probability of missingness of a variable is dependent on its value [1].

Since missing data are commonplace, several methods exist which address their ensuing problems. The most straightforward approach to dealing with missing data is simply to use only rows of the data that are not missing any values. This approach, commonly referred to as the “complete case” or “list-wise deletion” approach, is useful when the amount of missing data is small (some recommend no more than 5% of the data be missing), but inappropriate when there is substantial missing data [2–4]. In the latter situation, a complete case approach is likely to be both underpowered and biased. A popular approach to mitigate these issues, multiple imputation (MI), involves replicating the analytic dataset *m* times and then in each replicated dataset filling in missing values with a stochastic “reasonable guess” (imputation) of what the true value might be. Due to the stochastic component, each of the *m* datasets is filled in with slightly different guesses. A variety of imputation methods exist, two of which will be analyzed in this paper: predictive mean matching (PMM) and random forest (RF) imputation [5]. Multiple imputation is often paired with chained equations, also known as the fully conditional specification, which specifies the multivariate imputation model one variable at a time. Starting with an initial simple imputation (e.g., mean imputation), multivariate imputation by chained equations uses a sequence of iterations from the conditionally-specified models to generate imputed values that reflect relationships in the data. This technique can be generalized well to both continuous and categorical data [6].

PMM imputation is particularly useful for continuous variables and is conducted by estimating a value for a missing data cell with multiple regression based on other columns that are not missing data. For a given “modeled” column, observations with similar predicted values (of the same column) are placed together into small groups regardless of whether any data were initially missing. Then, the missing observations are randomly assigned the true values of the rows from their group which were not missing the modeled column. This process allows realistic values to be used in imputations that certainly exist on the domain of the data as well as maintain some variability [5, 7]. RF imputation is a powerful non-parametric method which involves building a random forest for each variable with missing values and using the results of that random forest to impute data. Typically, a sample of non-missing data will be sampled with replacement to create a classification or regression tree. This process will be repeated multiple times on bootstrap resamples, creating a variety of trees, which results in a bootstrapped random forest based on observed data. With this random forest, one can randomly select observed values from the terminal nodes of each of the trees in the forest to replace the missing data. Multiple random samples from these terminal nodes can create multiple imputed datasets [8]. Random forests can conveniently handle both categorical and continuous data and will often perform well in the presence of interacting or non-linear relationships.

Regardless of the exact imputation method, once all *m* copies of the original dataset have had their missing data stochastically filled in with imputed data, statistical analyses are performed on each imputed dataset to produce *m* results pertaining to the research question at hand. The final step of the MI process is to then pool the *m* results together and treat the combined outcome as the result [9]. When the statistical analysis of the imputed datasets involves an estimate or test statistic that is normally distributed, the pooling of results can be accomplished using a process called *Rubin’s rules*. To obtain the combined estimate, one can simply take the mean of the estimates over the *m* analyses. Obtaining the combined variance involves a calculation with the variance of the estimate across imputations with the average variances of the estimate within each imputation. Then, one can draw final conclusions with a Wald test based on the mean estimate and the combined variance [9, 10]. However, this pooling process is designed to work only with estimates that are normally distributed; when presented with estimates or test statistics that are non-normally distributed, one must use alternative methods to pool results from *m* multiply imputed datasets.

One such instance of complex models that do not have normally distributed estimates or test statistics are generalized additive models (GAMs). GAMs are useful for their ability to fit a line through data with varying “curvy-ness” in a more efficient and elegant manner than other traditional polynomial functions. Although most often employed for purposes of prediction and description, GAMs will sometimes be used for hypothesis testing and inference; our later applications concern situations where a flexible model is desired alongside hypothesis tests. Several fitting procedures exist to estimate the components involved in GAMs, most of which have a penalty term which can optimize model fit while protecting against overfitting. However, with such flexibility comes the cost of no longer having interpretable slope estimates or normally distributed test statistics. Thus, GAM parameters have no meaningful interpretation and cannot be combined with straightforward pooling methods. In general, beyond plotting a GAM, the only way to examine the importance of a GAM association numerically is to examine its effective degrees of freedom along with an approximate F statistic. These approximations are detailed in Wood [11, 12] and can provide a *p*-value which, if small, suggests that the relationship between a predictor in the GAM and the outcome is not a perfectly horizontal line. Wood’s approximations for these F statistics and effective degrees of freedom (and their corresponding *p*-values) are implemented in the R package mgcv [11]. Therefore, rather than attempting to apply normal pooling rules to these non-normal statistics, we suggest that additional methods of pooling are necessary when using GAMs together with multiple imputation.

We set out to examine several methods of pooling GAMs after MI based on their F statistics, effective degrees of freedom (edfs), and *p*-values. A simple combination method to pool GAMs after MI, and the focus of this study, is to take the median of all imputed within-imputation GAM *p*-values as the pooled *p*-value measuring the strength of evidence for the association. Eekhout, Wiel, and Heymans [13] aptly named this method the median *p*-value (MPV) rule, and they investigated its utility in determining the significance of categorical predictor variables in logistic regression models after MI. They demonstrated that in null models, the Type I error rate of results based on the MPV rule is only slightly inflated. In situations where the alternative hypothesis is true, the MPV rule performs equal to or better than other conventional methods. Several other methods for combining GAMs after MI are applicable as well; the most complex of which is the D2 method outlined by van Buuren [5] and originally proposed by Rubin [14], which involves combining test statistics in such a way to test against a "pooled" F distribution. Another method, the Cauchy combination test proposed by Lui and Xie [15], involves transforming then summing *p*-values into a joint Cauchy-distributed test statistic which can then be compared against the Cauchy cumulative distribution function. The Cauchy combination test was introduced in the context of genomic data, and to our knowledge has not been applied in the context of MI. Finally, we will investigate some alternative ad-hoc approaches described in the following section.

Our primary motivation in this work is to demonstrate the viability (or lack thereof) of the MPV rule in situations where MI and GAMs are used in conjunction. Such an exceptionally straightforward pooling method, if empirically valid, would be a welcome and cogent solution to this complex problem. In this work, we compare the empirical performance of the MPV in terms of power and type I error control relative to a suite of possible alternatives, using simulations and case studies. Our simulation studies were adapted from Friedman [16] to fit GAMs on multiply imputed data to variables which have varying degrees of "true" signal (including one variable with a true signal of zero). We additionally vary the imputation methods (PMM and RF), the missingness mechanisms (MCAR, MAR, and MNAR), and the outcome type (continuous/dichotomous). We then apply our proposed MPV rule and its competitors in two case studies, one examining the effect of home elevation (i.e. meters above sea level) on a six-minute walk distance for patients with pulmonary arterial hypertension (PAH), and the other investigating the extent to which C-reactive protein is associated with the risk of mechanical ventilation for patients infected with the novel Coronavirus disease (COVID-19).

## Methods

### Proposed multiple imputation pooling methods

In this section, we describe the various methods we examined in this study. The first, D2, involves pooling F-statistics from the *m* GAM fits and then comparing a pooled version of the F-statistic to an F-distribution whose numerator and denominator degrees of freedom are based upon the variance of F-statistics across imputations, the effective degrees of freedom from each GAM, and the number of imputations. Further details regarding this method can be found in van Buuren [5]. This D2 method is the most complex of the approaches evaluated in this study, and while understandable, it is not immediately intuitive. Some functionality is provided in R for D2 in the "mice" package [17], but the existing implementation is not applicable in the GAM context without custom programming. Furthermore, a component of the D2 method involves dividing the average test statistic across imputations by the number of parameters in the model; however, the number of parameters in GAMs are not fixed between models that are run on different imputations, so the correct denominator to use in the equation is unclear. Therefore, we evaluate two versions of the D2 method, one in which the average test statistic across imputations is divided by the average number of parameters across imputations (D2) and the other which is the average of the quotients of each test parameter divided by the number of parameters within imputation models (Alt. D2), as shown below:

$$D2:\frac{mean(test\;statistics)}{mean(number\;of\;parameters)}$$

$$Alt.\ D2:mean{\left(\frac{test\ statistics}{number\ of\ parameters}\right)},$$

where means are taken across *m* imputations

The second method we investigated is the Cauchy combination test, which deals only with *p*-values (as do all other considered combination methods). The Cauchy combination test is conducted by summarizing *p*-values from all imputations using the formula below into t_0_, then comparing this statistic against a standard Cauchy distribution:

$${t}_{0}= \sum_{i=1}^{m}\mathit{tan}\{(.5-{p}_{i})\pi\}$$

$$p\text{-value}= \frac{1}{2}-(\mathit{arctan}\ {t}_{0})/\pi$$

This method requires fewer calculations and is easier to implement than the D2 method. The Cauchy combination method was originally conceived to assess significance among large numbers of tests in genome-wide association studies and was designed to be most accurate for small *p*-values. The fatter tails of the Cauchy distribution reduce the effects of correlation between *p*-values of similar tests, and in our case, tests from multiply imputed datasets are likely to be correlated [15].

Finally, our proposed solution, the MPV rule, is simply to take the median *p*-value across the *m* GAM results. Ultimately, in this context, the analyst needs a singular summary measure, and the median of all of the *p*-values is a concrete (if ad-hoc) method that has been shown to work in other situations [13]. The median *p*-value across all GAMs captures the central tendency of all individual *p*-values in a robust manner. We also explore the empirical properties of several other ad-hoc methods, including the mean *p*-value rule (identical to the MPV but with a different measure of central tendency), a single imputation approach (simply using results from a single imputed dataset, avoiding any pooling procedures) and a complete case (list-wise deletion) approach.

### Simulation

Data for the simulation was generated using a model adapted from Friedman ([16], p. 37):

$$f\left(x\right)=10\mathit{sin}({x}_{1}{x}_{2}\pi )+20{({x}_{3}-.5)}^{2}+10{x}_{4}+5{x}_{5}+0{x}_{6}+\varepsilon$$

where

$$\varepsilon \sim N\left(0,9\right)$$

and

$${x}_1,\dots,{x}_6\sim unif\left(0,1\right)$$

We chose this true generating model due to its reasonable number of variables, its variety of beta parameter sizes, and its diversity of functional forms (trigonometric, polynomial, and linear). The GAM approach should feasibly be able to approximately capture the relationship between each covariate (except for x_6_) and the response, while results pertaining to x_6_ can be considered as evaluating the type I error rate (since the true relationship between x_6_ and y is a horizontal line, i.e., the null is true). Each covariate was generated from an independent uniform(0, 1) distribution. While the independence assumption is somewhat unrealistic and will limit imputation quality, it allows us to examine the power of each covariate’s relationship with the outcome more precisely. A similar model was utilized in simulations with a binary outcome; a standardized version of this formula produced a series of normally distributed random variables which were then transformed into a probability through a logit link and fed into a binomial process. This results in a binary outcome for which the log-odds of success are related to each covariate according to the same functional form as above.

**Fig. 1:**
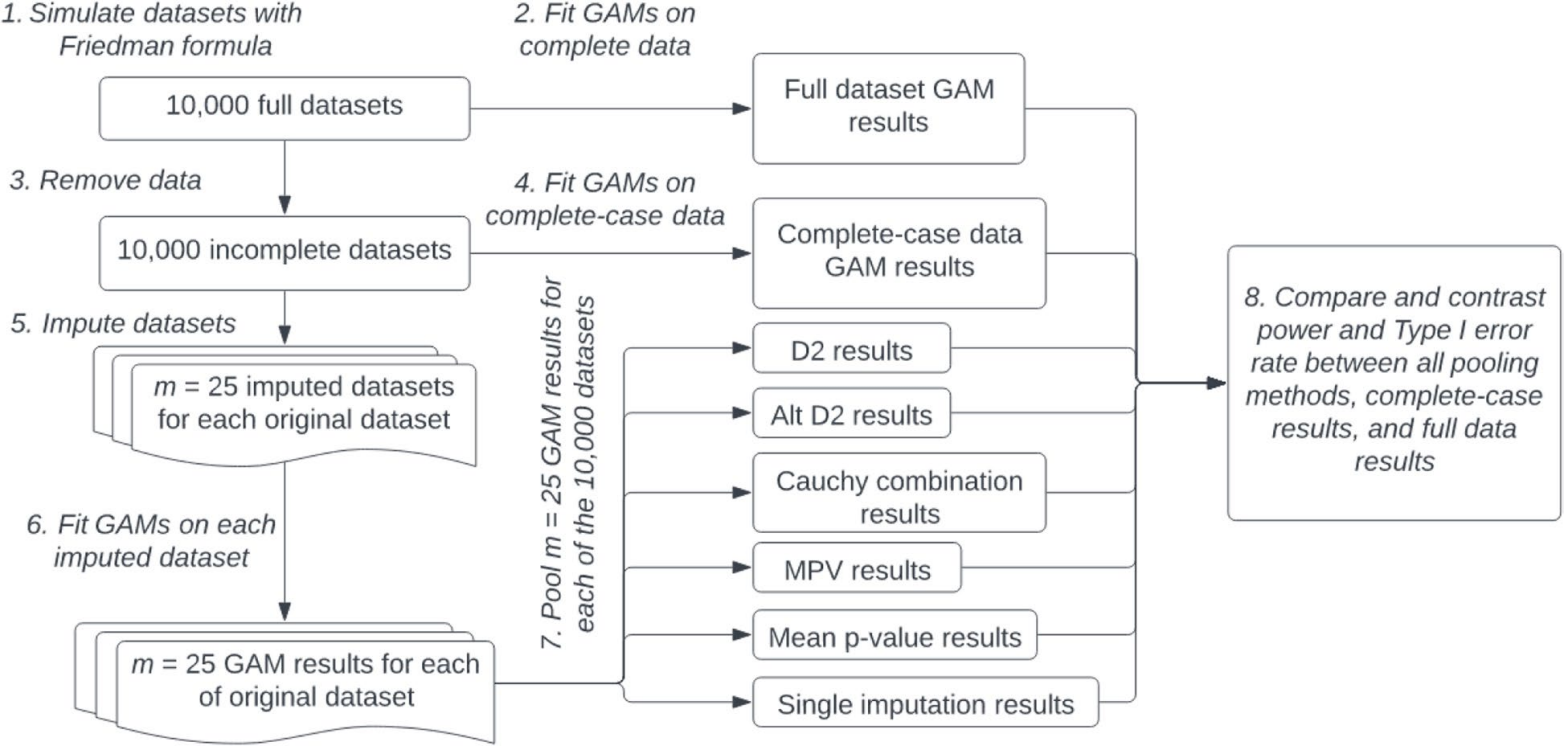
Simulation study flow chart

Given a missingness mechanism and outcome type, each simulation study was carried out using the steps below (also outlined in a flow chart in Fig. 1):

1. The Friedman generating model was used to simulate *S* = 10,000 datasets.
2. GAMs were fit to all full datasets wherein each covariate was modeled with its own smoothed term to produce our primary “full-data” (i.e. “gold-standard”) benchmarks. Because GAMs can vary in their number of basis functions, we specified that each fit be limited to a maximum of 10 basis functions to maintain model consistency across imputations and simulations. Note that for binary outcomes, GAMs were fit using a binomial-family model with a logit link.
3. Next, using the mice package [17], we simulated missingness within each dataset at a rate of 35% under the prespecified missing data pattern. That is, after simulating missingness, roughly 65% of rows in a dataset had no missing data, while the other 35% were missing data for at least one variable. Description and verification of the procedure to simulate these missing data patterns is outlined in Schouten, Lugtig, and Vink [18].
4. As a second benchmark, GAMs were fit using list-wise deletion (which reduced the sample to 65% of its original size) to allow for comparison of methods to the complete case approach (arguably, the simplest possible approach to missing data).
5. Each dataset was then multiply imputed using chained equations with *m* = 25. Default options were utilized under both random forest and predictive mean matching approaches. Imputation quality is described by Figure A7 in Additional file 1: Appendix.
6. We ran similar GAMs (as in steps 2 and 4) on each of the *m* imputed datasets.
7. We implemented the D2, Cauchy combination, MPV, and mean *p*-value rules to pool results on the GAMs from step 7.
8. Pooled *p*-values were compared to a significance level of 0.05 to compute power to detect the associations for x_1_ through x_5_ and to calculate Type I error rates for x_6_. These were then compared to both the full-data results (gold-standard) and complete case results.

These steps were repeated for normal and binary outcome data and under MCAR, MAR, and MNAR missing data patterns, which resulted in six total simulation studies. Attention is primarily paid in this paper to MAR data, but results under the MCAR and MNAR framework are also presented in Additional file 1: Appendix.

Preliminary analysis revealed that the default GAM fitting option, generalized cross-validation (GCV), often produced results with a much higher type I error rate than the anticipated 5%. A short comparison of model fitting techniques revealed that between maximum likelihood (ML), restricted maximum likelihood (REML), and GCV, the ML method produced type I error rates closest to, although still slightly higher than, the expected rate of 5%. This finding is consistent with writings from Wood [11], where GCV is found to produce less accurate results than ML, and from Wood [19, 12], where it is argued that GCV is more at risk than ML or REML of global optimization failure, which in turn under-penalizes over-fitting and leads to a higher Type I error rate. Therefore, all models in the simulation study utilized ML for GAM fitting; however, all three fitting methods are examined and compared in our first application. Finally, we repeated the simulation studies with cubic B-splines in lieu of GAMs to evaluate and compare the performance of the MPV using an alternative semi-parametric spline model for normal outcomes.

## Results

**Fig. 2:**
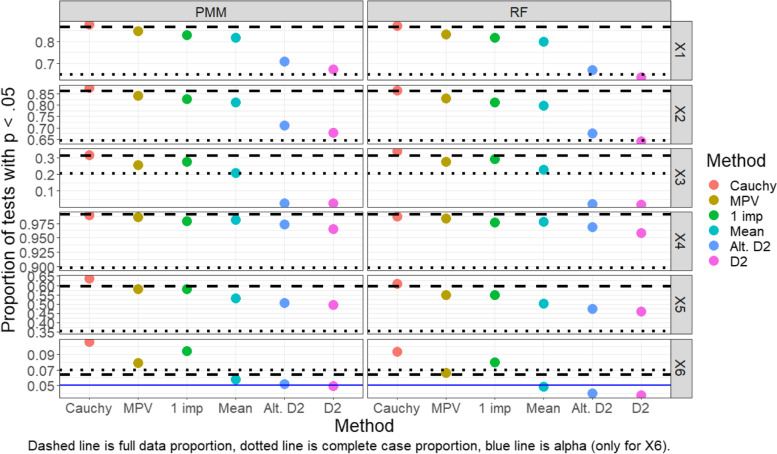
Proportion of tests that rejected the null hypothesis (normal outcome, MAR, GAM)

**Fig. 3:**
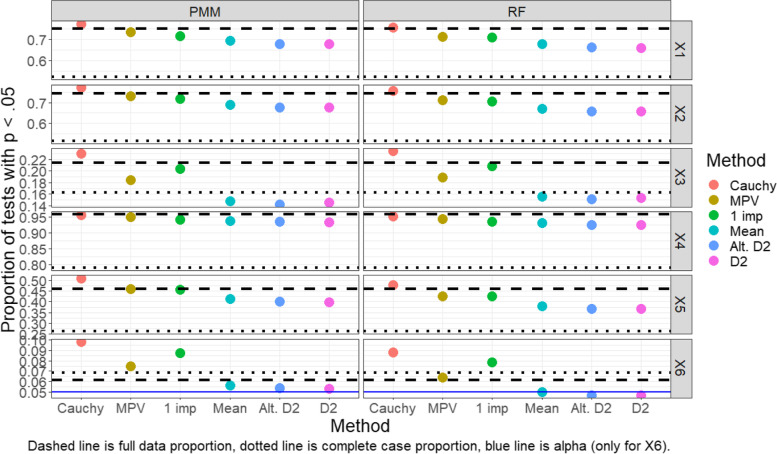
Proportion of tests that rejected the null hypothesis (binary outcome, MAR, GAM)

Results for each of the pooling methods, as well as the complete case analysis and full data analysis, are shown in Fig. 2 (normal outcome) and Fig. 3 (binary outcome).

In Fig. 2, we find the pooling methods have a somewhat consistent order in regard to proportion of tests rejected at *p* < 0.05 across imputation methods and variables, with the proportion of rejections being highest for the Cauchy combination method, followed by the MPV rule, a single imputation approach, a mean *p*-value rule, and then the two D2 methods. The exceptions to this ordering occur for the third and sixth covariates, for which single imputation rejects a larger proportion of tests than the MPV rule.

The Cauchy combination rule seems to be the highest-powered approach for pooling; in all situations it has the highest proportions of tests with *p* < 0.05, sometimes suspiciously finding more significant findings than the “gold standard” models run on the full data. However, for the x_6_ variable which has a null effect, the Cauchy combination rule significantly over-rejects tests compared to other methods. Curiously, almost all methods over-rejected tests (i.e., rejected tests at a rate higher than 5%) for the x_6_ variable, including the full data analysis and complete case analysis. In terms of power, the MPV rule performs only slightly worse than the full data analysis for variables x_1_ through x_5_ and performs much better than the complete case analysis in most settings. On the other hand, both D2 methods perform rather poorly (e.g., low power) in most situations compared to the other approaches, although they still typically perform better than the complete case analysis. The D2 methods additionally have low Type I error rates (rates that are much lower than those for the full data or complete case approaches).

Findings for the binary outcome data under a MAR framework are very similar, as shown in Fig. 3. Additionally, findings regarding the capability of these pooling methods were similar in simulations conducted under MCAR and MNAR frameworks (see Additional file 1: appendix). A point of peculiarity is that in both the normal and dichotomous outcome simulations, the Type I error rate in the perfect, full-data data case deviates further than anticipated from 5%. We conjecture that the *p*-value distribution even in the full-data case is biased due to the inherent variability in the smoothing parameter selection that is not being accounted for when testing the null hypothesis. However, as mentioned previously, these GAMs were fit with a ML approach which in preliminary analyses had lower Type I error rates than REML or GCV. So, although the Type I error rate is not as low as one might hope for in the perfect, full-data data case, we have utilized what we believe to be currently the most conservative method of all fitting options.

**Fig. 4:**
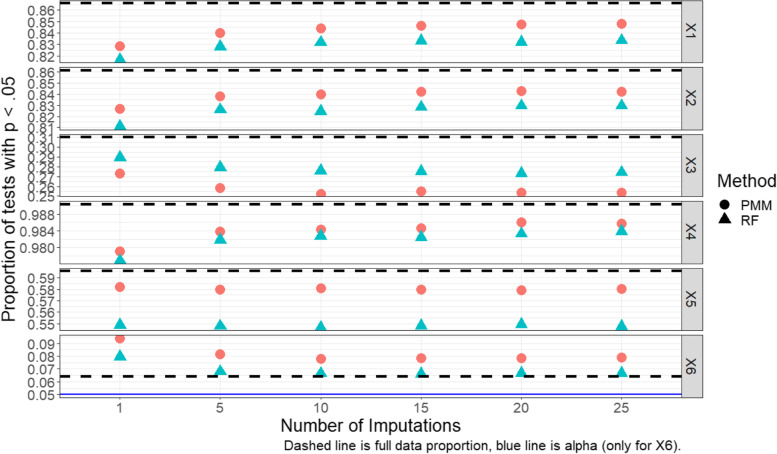
MPV rule performance by number of imputations (normal outcome, MAR, GAM)

A natural follow-up question that arises for the MPV is: how many imputed datasets are necessary to achieve satisfactory performance? To shed light on this, we illustrate the proportion of tests with *p* < 0.05 using the MPV and several choices of the number of imputed datasets in Fig. 4. Generally, the performance of the MPV rule improved with an increased number of imputations; as the number of imputed datasets increased, power increased for x_1_, x_2_, and x_4_, and remained constant for x_5_, while the type I error rate decreased toward the full-data level. Improvements were greatest for low number of imputations and tapered off after 10 imputations. Strangely, this was not the case for x_3_, where the MPV rule’s power decreased with additional imputations. This is because both PMM and RF have difficulties capturing the nature of the x_3_ relationship to Y (see Additional file 1: Appendix Figure A7), particularly PMM, since the relationship is non-linear in such a fashion that the linear relationship is biased toward the null (U-shaped).

PMM imputation seemed to yield results more consistent with the full data approach than RF imputation, with the exceptions of x_3_ and the null variable x_6_, where the type I error rate was higher for PMM imputed data. As stated above, the better performance of RF over PMM for x_3_ is likely due to RF imputation’s ability to better identify and model non-linear relationships.

**Fig. 5:**
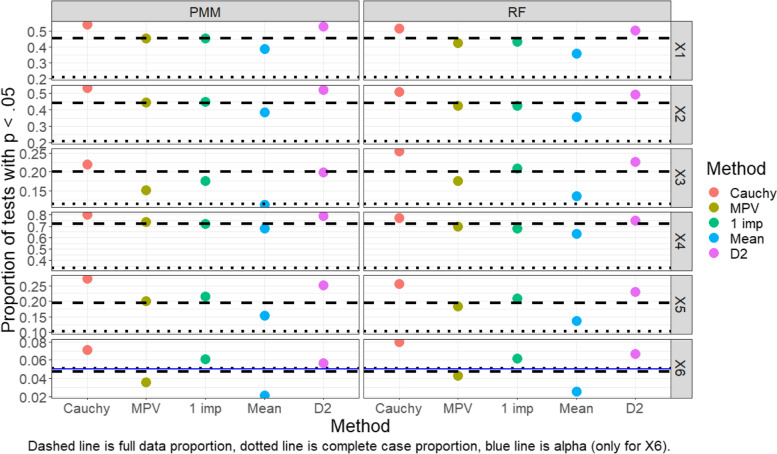
Proportion of tests that rejected the null hypothesis (normal outcome, MAR, B-spline)

In addition to GAMs, the effectiveness of the MPV in comparison to other methods was also examined on B-spline models. B-spline models, like GAMs, are a technique for fitting a smooth curve through data, where the curviness of the line is possible due to the combination of “knots” being placed along the x-axis and constrained polynomial terms. In our investigation, we examined cubic B-spline regression models with 10 degrees of freedom with knots placed at seven internal equally-spaced quantiles of the covariate. B-splines were examined only with normal data, and inference was conducted using likelihood ratio tests (LRT). Figure 5 presents results for B-spline models on normal data with a baseline MAR missing data structure. The alternative D2 method was not used in this analysis, as the number of degrees of freedom in the case of B-splines is more tractable.

**Fig. 6:**
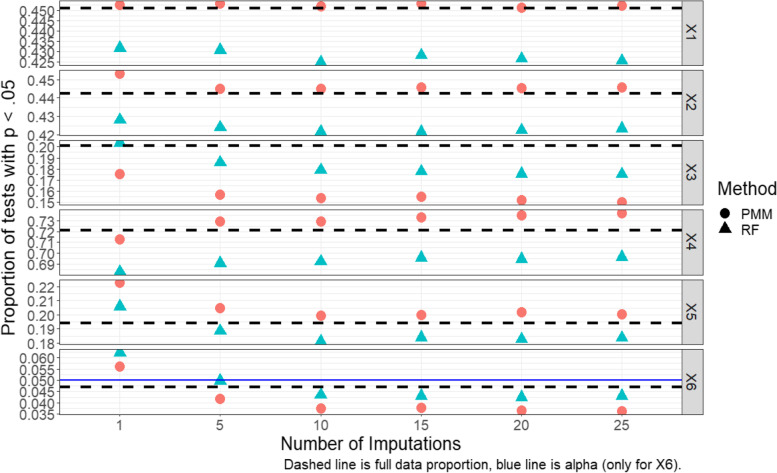
MPV rule performance by number of imputations (normal outcome, MAR, B-splines)

Again, Fig. 5 shows that the MPV performs comparably well in terms of power for covariates x_1_ through x_5_. Now, we also see a satisfactory performance of MPV for the null case (x_6_), in which, the MPV rule is closest to the expected Type I error rate while remaining slightly conservative. Further B-spline results are shown in the Additional file 1: Appendix for MCAR and MNAR missing data patterns. Figure 6 shows that the performance of the MPV generally improves or stays steady with additional imputations when used with LRTs of B-spline models, with the same exceptions as GAMs with the x_3_ variable.

**Fig. 7:**
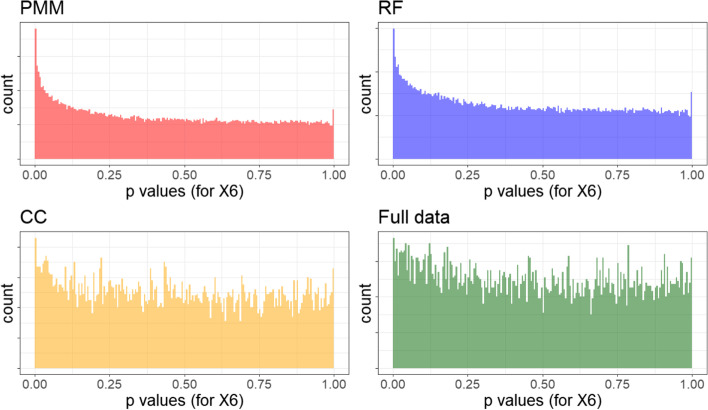
Distribution of *p*-values for null effect with GAMs

**Fig. 8:**
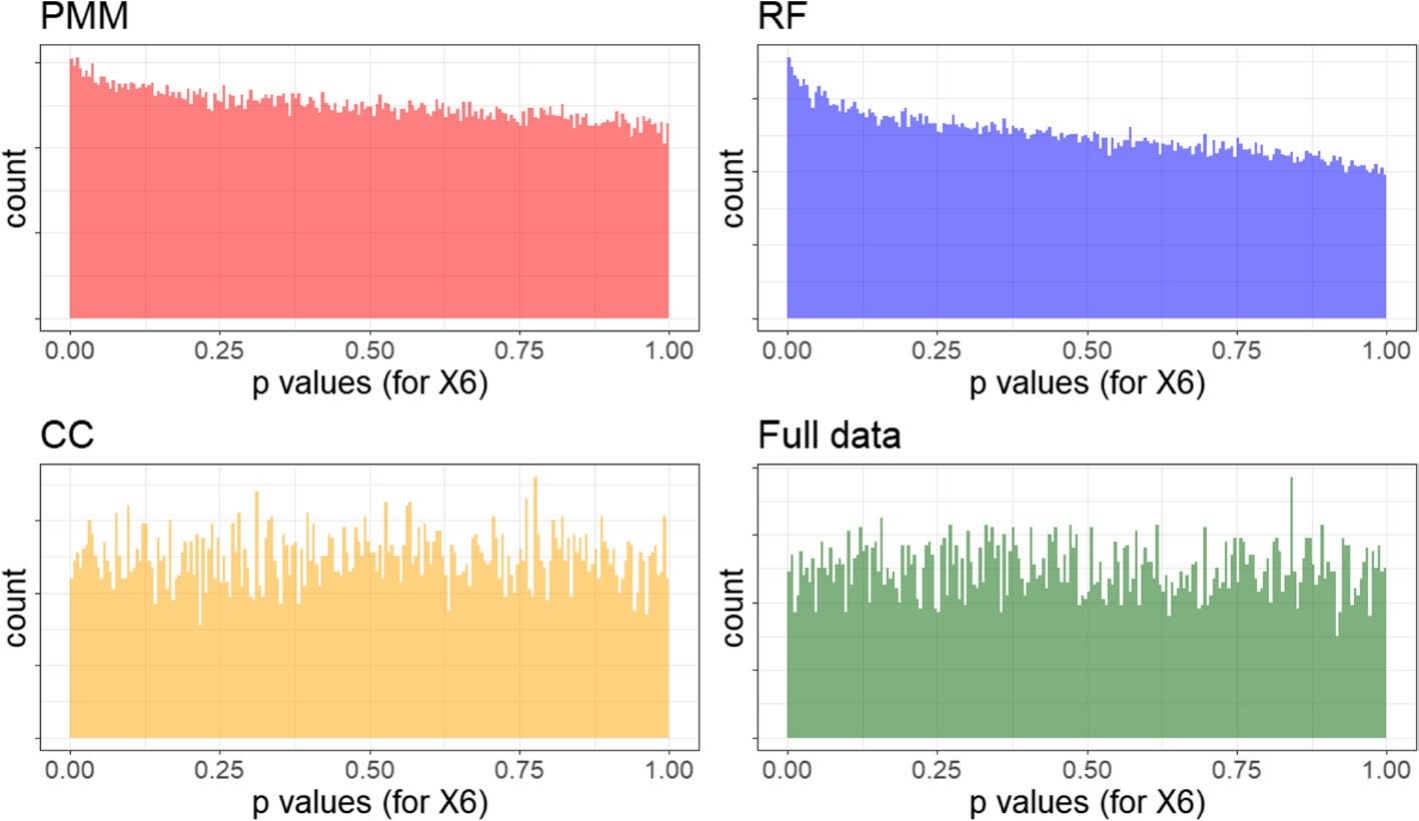
Distribution of *p*-values for null effect with B-splines

It is clear we observe bias away from the null in both B-spline and GAM settings. To investigate this, we plot the *p*-values for the null effect of x_6_ with the GAM results Fig. 7. We see that not only are PMM and RF *p*-values for null effects non-uniform (with a heavy lean towards smaller *p*-values), but the complete case and full data situations likewise are left-heavy. Although we found that the type I error rate was better controlled for B-splines than for GAMs, similar histograms indicate that the B-splines still show evidence of bias away from the null after imputations (Fig. 8). However, the full data and complete case rejections of the null are near-uniform with rejection rates almost exactly at 5%, in contrast to the higher rates seen with GAMs. In conclusion, we have found that default imputation methods (PMM and RF) bias results slightly against the null, but also that GAM *p*-values are biased against the null regardless of imputation technique as demonstrated by the inflated type I error rates even in the full data analysis.

### Normal outcome application: six-minute walk distance based on elevation in PAH patients

A recent paper [20] examined the effect of home elevation from sea-level (based on ZIP code) on distance walked during six minutes in patients presenting with pulmonary arterial hypertension. The six-minute walk distance (6-MWD) is an important clinical metric used for evaluating progression of disease. Several variables in this dataset were missing non-negligible amounts of data and the study authors used multiple imputation via chained equations with *m* = 25 imputations to address the missing data problem, using predictive mean matching as their imputation method for continuous variables and logistic or multinomial regression for categorical variables.

**Fig. 9:**
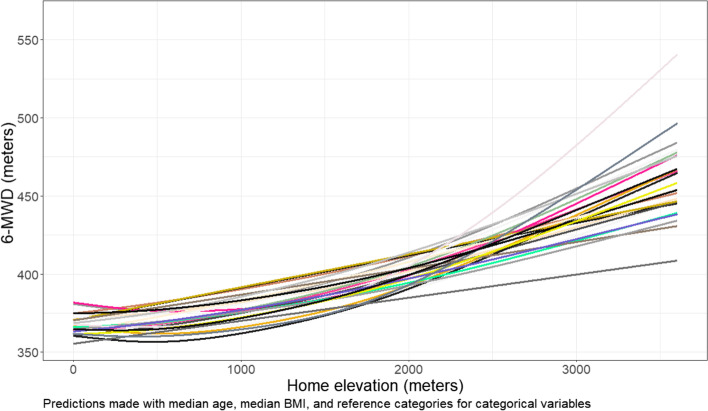
Effects of smoothed elevation on 6-MWD for each imputation

A central model of interest from this study was a GAM in which 6-MWD was modeled by various demographic and clinical covariates, as well as a smoothed term for continuous elevation. Elevation was provided based on patient home address. It was in this context that we apply the various rules of GAM combination across multiple imputations to evaluate how these methods perform on real-world data. Figure 9 shows the relevant covariate curves of 25 GAMs fit with ML to data from each of the *m* imputations. Although standard errors of the curves are not plotted for the sake of clarity, it is visibly evident how some curves might suggest a stronger relationship between elevation and distance walked than others.

**Fig. 10:**
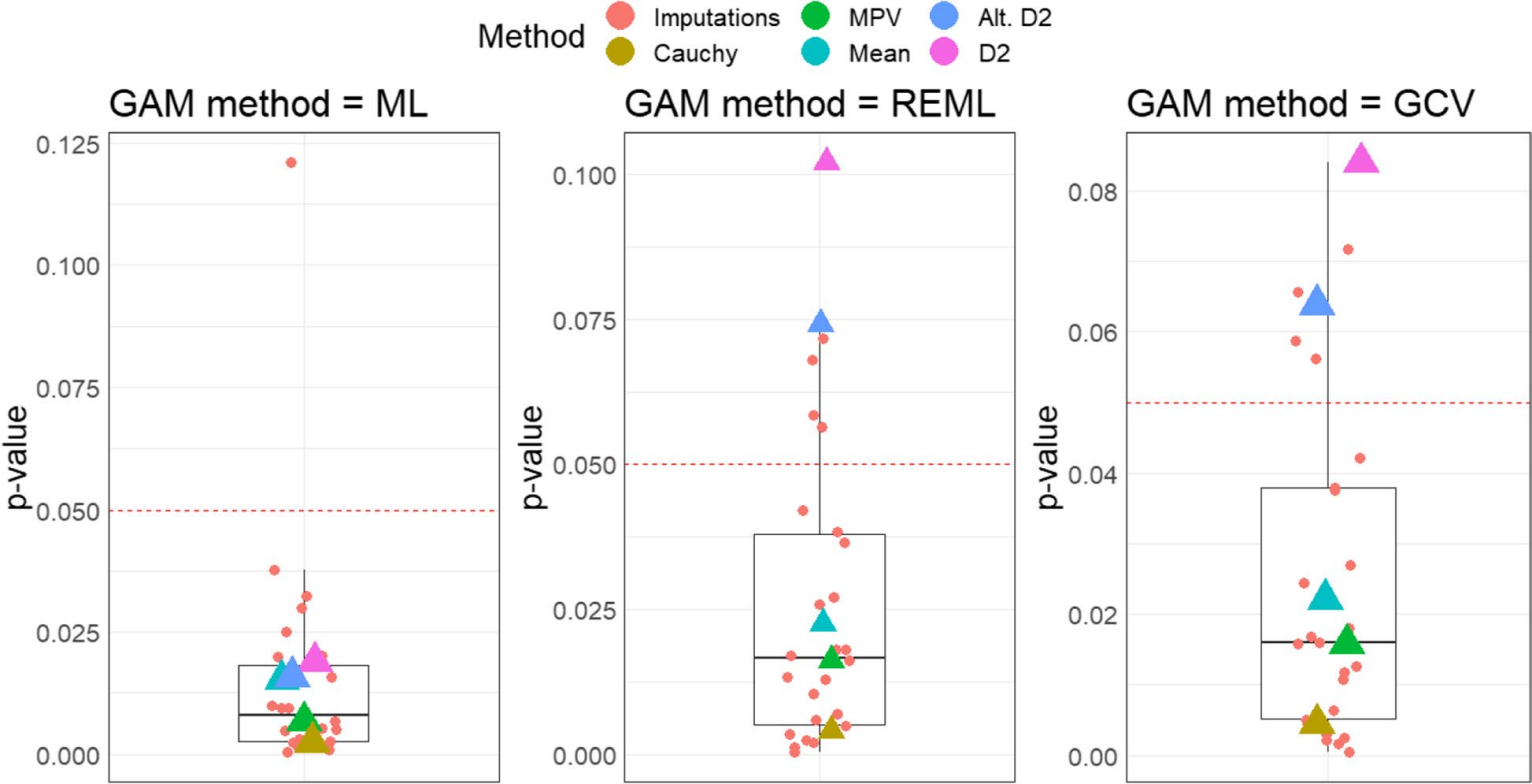
Imputed and combined *p*-values across fit methods

This heterogeneity across imputations is further demonstrated in Fig. 10, which plots all *p*-values from each imputation’s GAM as well as the results from all investigated pooling methods. The three panels represent three GAM fitting methods: ML, REML, and GCV, the last of which is the default GAM fitting method. The motivation for conducting GAMs with all three fitting methods was derived from preliminary concerns about inflated Type I error rates with the GCV approach.

For all model fitting options, the MPV approach yielded a *p*-value as anticipated – in the middle of all single imputation models' *p*-values. The Cauchy combination method gave smaller *p*-values than the other combination methods, while the D2 method and alternative D2 method produced *p*-values on the high end, if not outside the range, of the single imputation *p*-values. In this application, the selected pooling method could clearly impact the results of the study if the authors leaned heavily on the alpha = 0.05 criteria for statistical significance of *p*-values.

### Binary outcome application: Intubation risk by C-reactive protein level

Our second application uses data from Peterson [21] and Windham et al. [22] which modeled the risk of intubation for hospitalized patients infected with COVID-19 based on patient characteristics including C-reactive protein (CRP) levels, age, body mass index (BMI), race, sex, lactate dehydrogenase, and additional clinical measures. Limited clinical insight of risk factors for COVID-19 complications early in the pandemic necessitated the need for flexible modeling without restrictive parametric assumptions. Therefore, a GAM with a logit link was chosen with a smoothed term for CRP level to maximize its predictive ability on intubation risk.

**Fig. 11:**
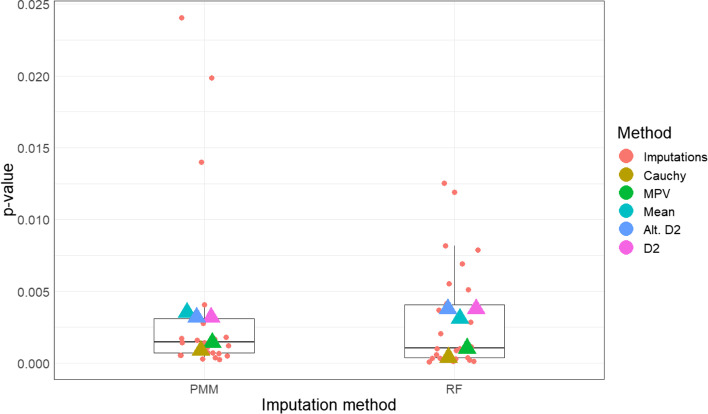
Imputed and combined *p*-values for CRP and intubation relationship

Missing data were non-negligible and dependent on observed patterns in the data. Therefore, we assumed a MAR framework and employed multiple imputation via chained equations with *m* = 25 imputations for analysis. As before, we examine the behavior of the MPV rule in this analysis in comparison to other pooling methods. ML was used in this application as the fitting method. Figure 11 shows the distribution of *p*-values after MI. The rank order of the combined *p*-values was similar between predictive mean matching and random forest methods. Again, we see that the Cauchy combination rule results in the smallest *p*-value, while the D2 and mean *p*-value rules have larger *p*-values. Unlike the prior application however, all methods lead to the same inferential decision (using a 5% significance level), so the choice of pooling method is less consequential. It is worth noting that a listwise deletion approach yields a *p*-value of 0.098, much higher than all other methods. Even though there were only 8 missing values for CRP out of the original 158 participants, only 51 of participants had complete data for all covariates, so listwise deletion cuts the sample to only 32% of its original size. Hence, multiple imputation was necessary in this example to fully leverage the available data and to maximize power/precision.

## Discussion

When missing data arise, MI has proven to be a useful approach to minimizing the loss of power and bias that often accompany missing data. When using flexible modeling techniques on MI datasets such as GAMs, pooling methods are not always straightforward, especially when test statistics or parameters are not normal and cannot be combined with Rubin’s rules. We have demonstrated that the MPV is a relatively valid, valuable, and straightforward way to pool results in comparison to other methods for estimation with GAMs or B-splines after MI. This result was found under MCAR, MAR, and MNAR missing data frameworks. This finding aligns with similar results from Eekhout, van de Wiel, and Heymans [13] in their application of the MPV to multi-categorical variables in logistic regression. Certainly, other methods have also demonstrated utility; if avoiding a Type II error significantly outweighs the cost of making a Type I error, then the Cauchy combination test is recommended. If the analyst is concerned with avoidance of Type I errors, then one of the D2 approaches or the mean *p*-value rule might prove useful. Aside from these cases, we conclude that true to its namesake, the MPV rule strikes an excellent middle ground by balancing decent power with a moderately controlled rate of false positives. Not only is the MPV an empirically helpful tool, but its simplicity facilitates its implementation in any software package.

The performance of D2 was somewhat poor in our simulation studies, but this is in line with what other empirical studies have found. In his description of the D2 method, van Buuren [5] describes that, in comparison to other combination methods, it is often underpowered since it utilizes less of the information that the data provides compared to other methods. However, in other simulations, the D2 method has performed too liberally, particularly with large sample sizes, large amounts of missing data, and few imputations [5]. Thus, the performance of D2 remains unpredictable.

There was a non-negligible amount of bias towards the alternative hypothesis in GAMs, even for models fit to the full dataset using maximum likelihood. This bias in the full-data models was attenuated using the B-spline LRT approach. However, since default options were used in the multiple imputation PMM and RF models, the imputation models were not correctly specified, which induced additional bias in both B-spline and GAM approaches when performed on imputed datasets (as seen in Figure A7 of the Additional file 1: Appendix). This observation indicates that a more flexible imputation model (e.g. one which utilizes splines) or a better-tuned imputation model would improve likely the performance of all methods by attenuating this remaining source of bias; this is a promising avenue for future research and additional simulation studies.

## Conclusions

In short, we have shown the MPV rule can be a useful analytic tool for pooling GAM or B-spline results after multiple imputation for statisticians who need a straightforward, accurate, and easily implemented pooling approach when dealing with missing data in MCAR, MAR, and MNAR situations. Not only does the MPV have adequate or superior power for detecting a variety of linear and non-linear relationships compared to its alternatives, but it also does a reasonable job of controlling the Type I error rate which seems to improve with the quality of the imputation model. While arguably the simplest method is to use the complete-case analysis and avoid the imputation procedure altogether, we have demonstrated that the MPV rule maintains simplicity while also leveraging all observed data to improve power and precision. Further research into the effectiveness of the MPV rule in additional settings such as nonparametric analyses, exact tests, and penalized regression may continue to expand upon on its utility in multiple imputation.

## Supplementary Information

**Additional file 1:** **Appendix.**

## Data Availability

The datasets used and/or analyzed during the current study are available from the corresponding author on reasonable request.

## Abbreviations

CRP: C-reactive protein
GAM: Generalized additive model
GCV: Generalized cross validation
LRT: Likelihood ratio test
MAR: Missing at random
MCAR: Missing completely at random
MI: Multiple imputation
ML: Maximum likelihood
MNAR: Missing not at random
MPV: Median *p*-value rule
PAH: Pulmonary arterial hypertension
PMM: Predictive mean matching (imputation method)
REML: Restricted maximum likelihood
RF: Random forest (imputation method)
6-MWD: Six-minute walk distance
