# Supplementary material for: Inference following multiple imputation for generalized additive models: an investigation of the median p-value rule with applications to the Pulmonary Hypertension Association Registry and Colorado COVID-19 hospitalization data
Source: BMC Med Res Methodol. 2022 May 21;22:148. doi: 10.1186/s12874-022-01613-w (PMC9123297; doi:10.1186/s12874-022-01613-w)
Supplement: Supplementary file 1 — Additional file 1: Appendix. [file 12874_2022_1613_MOESM1_ESM.docx]

**Appendix I: MCAR and MNAR missing data patterns for GAMs**


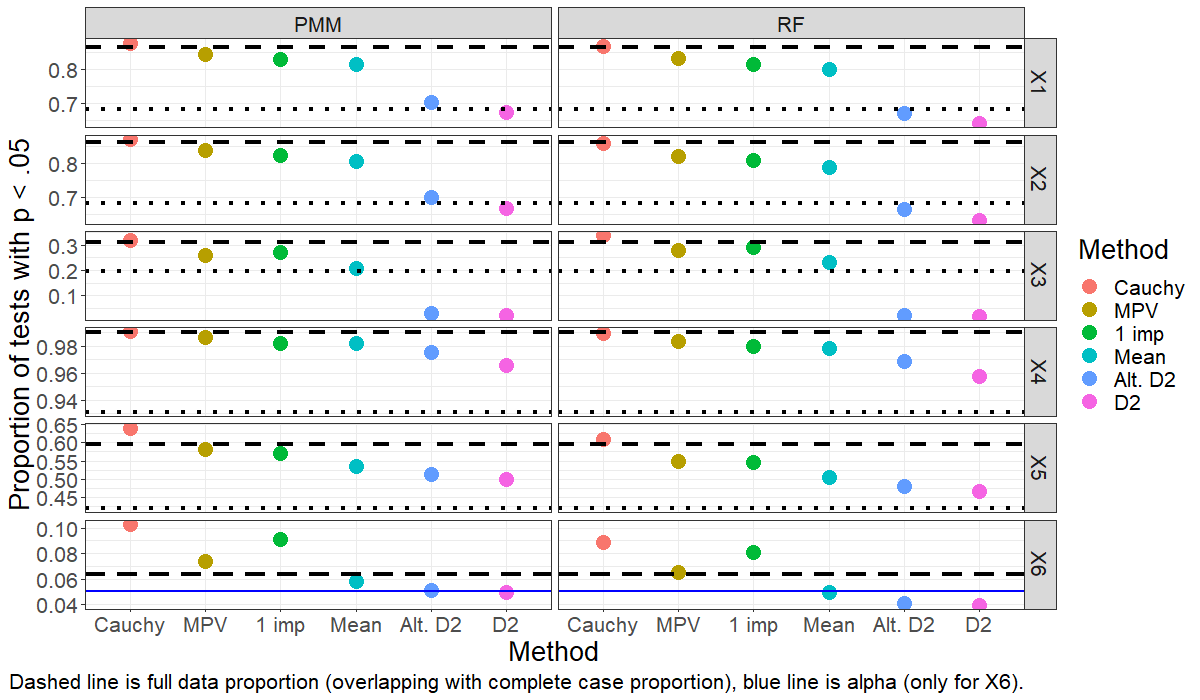
Figure A1. Proportion of tests that rejected the null hypothesis (normal outcome, MCAR, GAM)

Figure A2. Proportion of tests that rejected the null hypothesis (binary outcome, MCAR, GAM)


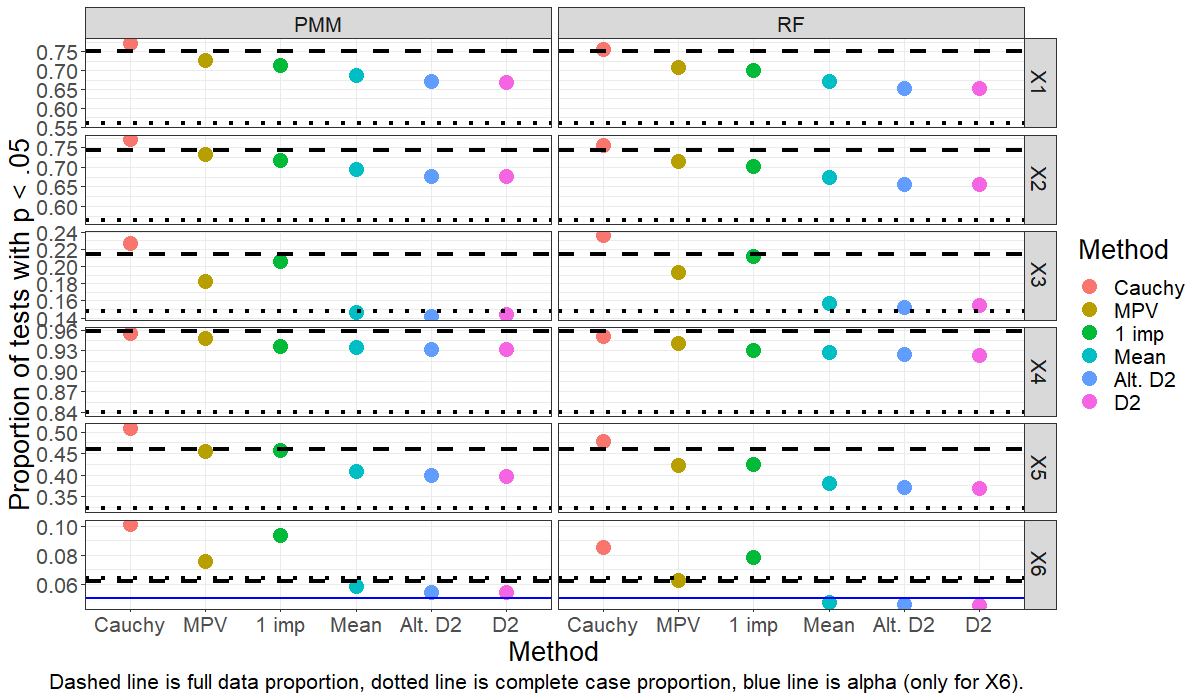


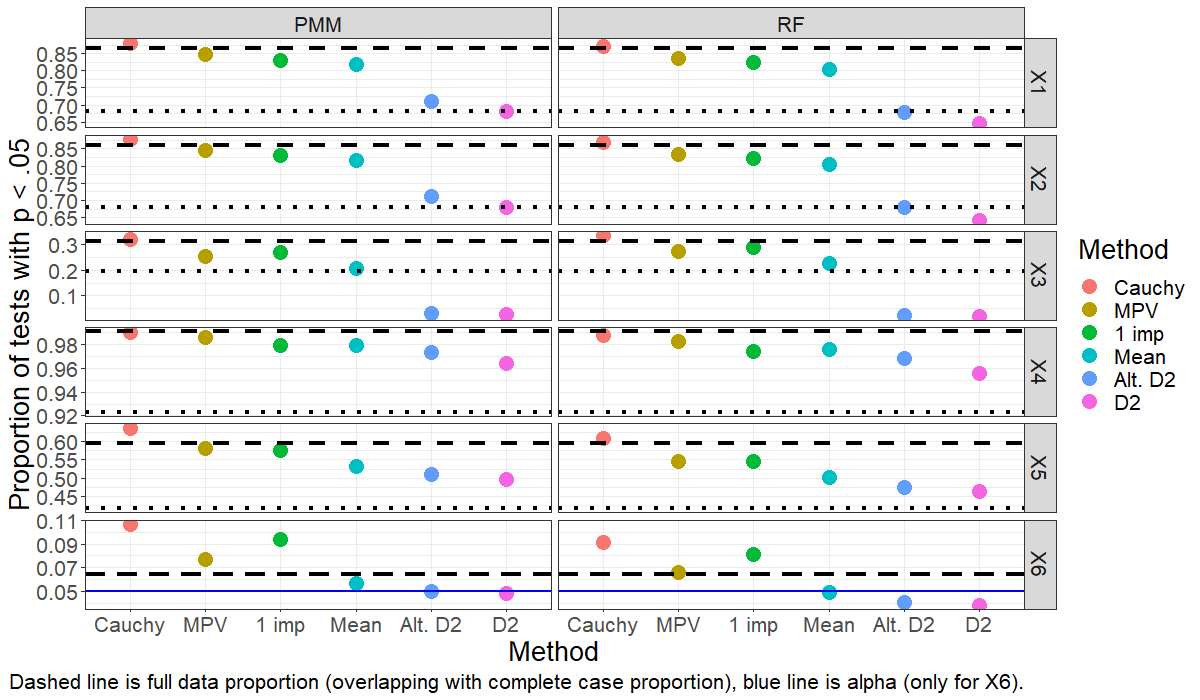
Figure A3. Proportion of tests that rejected the null hypothesis (normal outcome, MNAR, GAM)


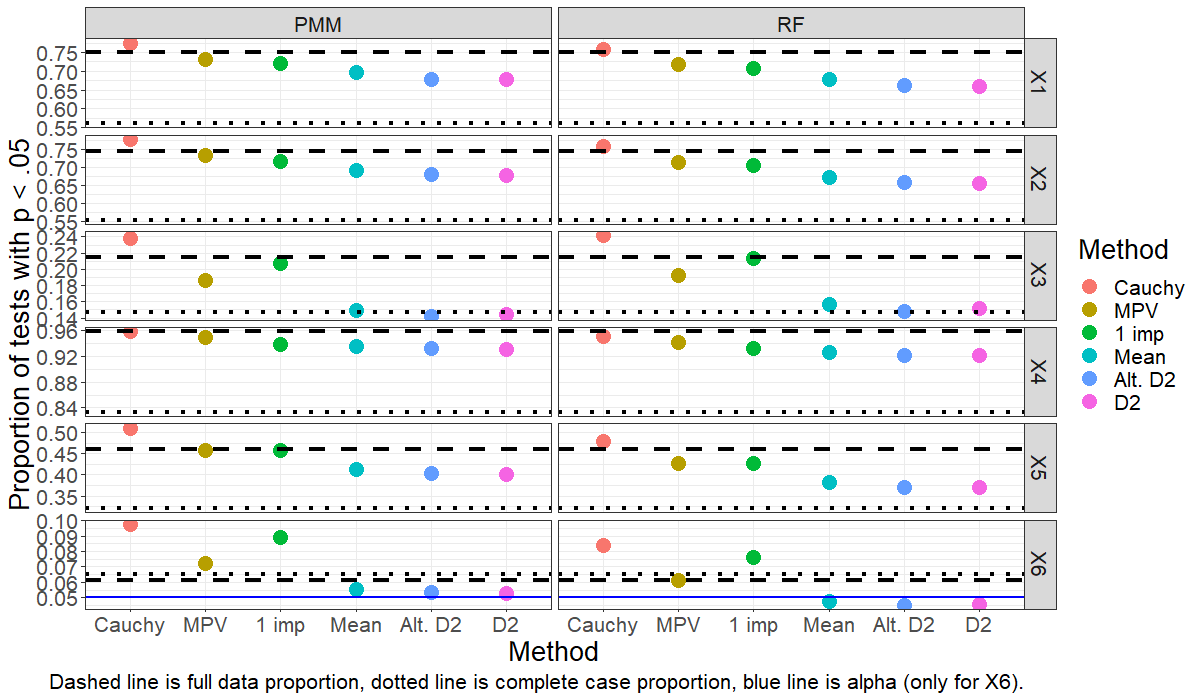
Figure A4. Proportion of tests that rejected the null hypothesis (binary outcome, MNAR, GAM)

**Appendix II: B-spline results**


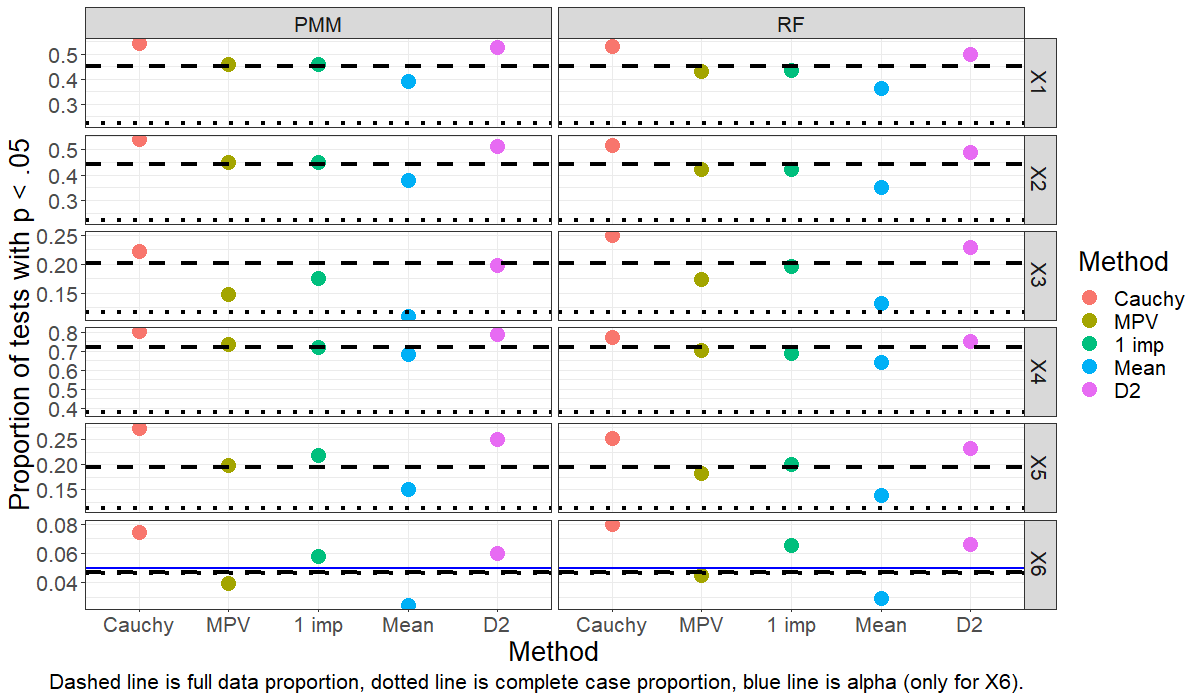
Figure A5. Proportion of tests that rejected the null hypothesis (normal outcome, MCAR, B-spline)

Figure A6. Proportion of tests that rejected the null hypothesis (normal outcome, MNAR, B-spline)


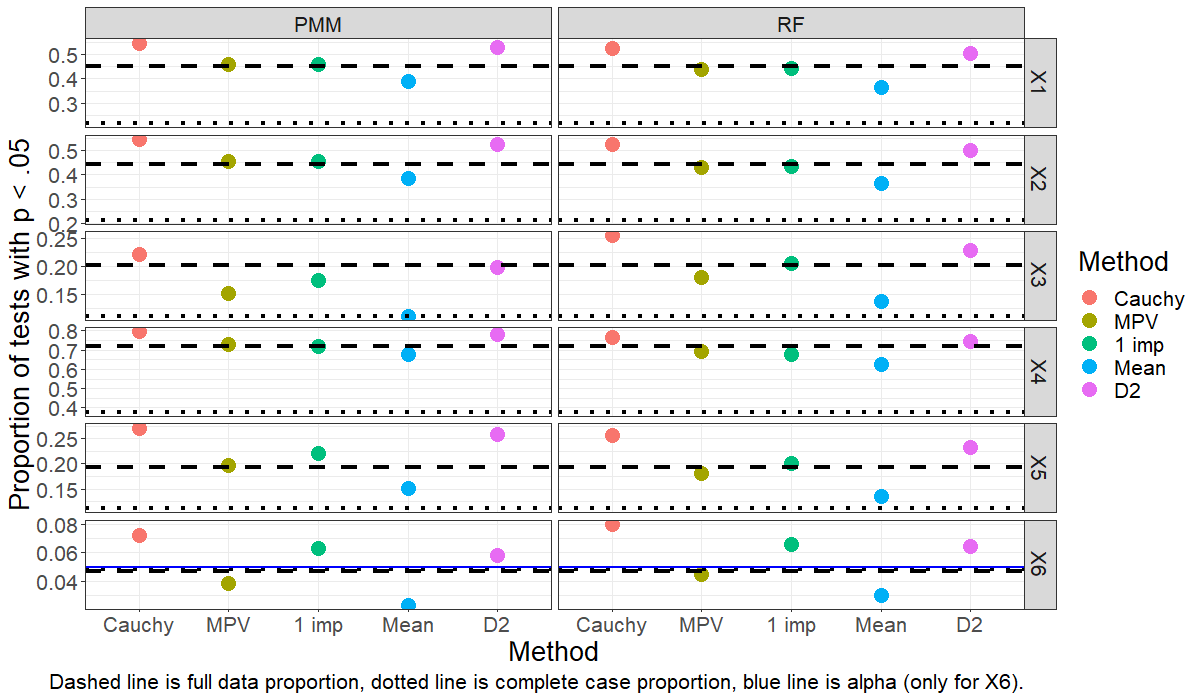


**Normal, MAR data imputation accuracy**

A visualization of the bias in the imputed values of Y under each method, shown alongside the true relationship. Each plot shows the expected value of y conditional on the given covariate, then mean-centered based on the covariate value. The black line represents the true relationship between Y and the covariate, while the red and blue lines show statistical contrasts of models where the outcome is the imputations for Y (PMM and RF respectively), and the covariates are GAMs on “true” values of all covariates. Statistical contrasts represent changes relative to the mean covariate value, while shading represents 1 SE on either side of the predicted value.


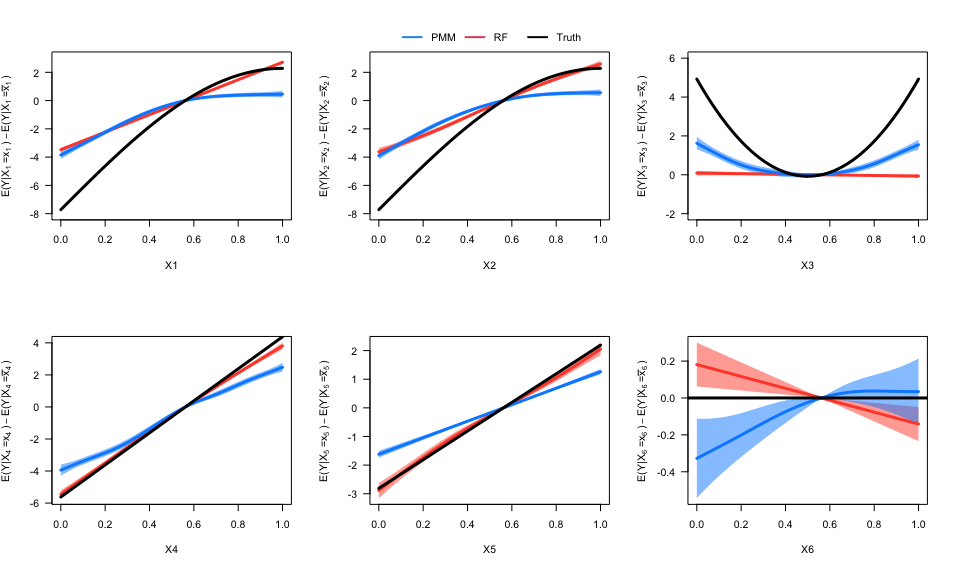
Figure A7. Imputation quality for MAR normal outcome data.
